# Supplementary material for: Unveiling the Bioactive Potential of the Invasive Jellyfish Phyllorhiza punctata Through Integrative Transcriptomic and Proteomic Analyses
Source: Biomolecules. 2025 Aug 4;15(8):1121. doi: 10.3390/biom15081121 (PMC12383608; doi:10.3390/biom15081121)
Supplement: Supplementary file 1 [file biomolecules-15-01121-s001.zip › Suplementary Material/FileS3/PEPFOLD4/XP_065065863_711/clusters.html]

 PEP-FOLD clusters


Download archive of all models  here   

|  |  |  |
| --- | --- | --- |
| # | file | sOPEP |
| # cluster 1 - Size: 10 |  |  |
|  | XP\_065065863\_711-model1 | -11.6131 |
|  | XP\_065065863\_711-model1.1 | -11.6131 |
|  | XP\_065065863\_711-model1.2 | -11.6131 |
|  | XP\_065065863\_711-model1.3 | -11.6131 |
|  | XP\_065065863\_711-model1.4 | -11.6131 |
|  | XP\_065065863\_711-model1.5 | -11.6131 |
|  | XP\_065065863\_711-model1.6 | -11.6131 |
|  | XP\_065065863\_711-model1.7 | -11.6131 |
|  | XP\_065065863\_711-model1.8 | -11.6131 |
|  | XP\_065065863\_711-model1.9 | -11.4753 |
| # cluster 2 - Size: 4 |  |  |
|  | XP\_065065863\_711-model2 | -11.5102 |
|  | XP\_065065863\_711-model2.1 | -11.5102 |
|  | XP\_065065863\_711-model2.2 | -11.5102 |
|  | XP\_065065863\_711-model2.3 | -11.5102 |
| # cluster 3 - Size: 9 |  |  |
|  | XP\_065065863\_711-model3 | -11.5014 |
|  | XP\_065065863\_711-model3.1 | -11.5014 |
|  | XP\_065065863\_711-model3.2 | -11.5014 |
|  | XP\_065065863\_711-model3.3 | -11.5014 |
|  | XP\_065065863\_711-model3.4 | -11.5014 |
|  | XP\_065065863\_711-model3.5 | -11.5014 |
|  | XP\_065065863\_711-model3.6 | -11.5014 |
|  | XP\_065065863\_711-model3.7 | -11.5014 |
|  | XP\_065065863\_711-model3.8 | -11.5008 |
| # cluster 4 - Size: 42 |  |  |
|  | XP\_065065863\_711-model4 | -11.4924 |
|  | XP\_065065863\_711-model4.1 | -11.4924 |
|  | XP\_065065863\_711-model4.2 | -11.4924 |
|  | XP\_065065863\_711-model4.3 | -11.4924 |
|  | XP\_065065863\_711-model4.4 | -11.4924 |
|  | XP\_065065863\_711-model4.5 | -11.4924 |
|  | XP\_065065863\_711-model4.6 | -11.4924 |
|  | XP\_065065863\_711-model4.7 | -11.4924 |
|  | XP\_065065863\_711-model4.8 | -11.4924 |
|  | XP\_065065863\_711-model4.9 | -11.4924 |
|  | XP\_065065863\_711-model4.10 | -11.4924 |
|  | XP\_065065863\_711-model4.11 | -11.4924 |
|  | XP\_065065863\_711-model4.12 | -11.4924 |
|  | XP\_065065863\_711-model4.13 | -11.4924 |
|  | XP\_065065863\_711-model4.14 | -11.4924 |
|  | XP\_065065863\_711-model4.15 | -11.4924 |
|  | XP\_065065863\_711-model4.16 | -11.4924 |
|  | XP\_065065863\_711-model4.17 | -11.4924 |
|  | XP\_065065863\_711-model4.18 | -11.4924 |
|  | XP\_065065863\_711-model4.19 | -11.4924 |
|  | XP\_065065863\_711-model4.20 | -11.4924 |
|  | XP\_065065863\_711-model4.21 | -11.4924 |
|  | XP\_065065863\_711-model4.22 | -11.4924 |
|  | XP\_065065863\_711-model4.23 | -11.4924 |
|  | XP\_065065863\_711-model4.24 | -11.4924 |
|  | XP\_065065863\_711-model4.25 | -11.4924 |
|  | XP\_065065863\_711-model4.26 | -11.4924 |
|  | XP\_065065863\_711-model4.27 | -11.4924 |
|  | XP\_065065863\_711-model4.28 | -11.4924 |
|  | XP\_065065863\_711-model4.29 | -11.4924 |
|  | XP\_065065863\_711-model4.30 | -11.4924 |
|  | XP\_065065863\_711-model4.31 | -11.4924 |
|  | XP\_065065863\_711-model4.32 | -11.4924 |
|  | XP\_065065863\_711-model4.33 | -11.4924 |
|  | XP\_065065863\_711-model4.34 | -11.4924 |
|  | XP\_065065863\_711-model4.35 | -11.4924 |
|  | XP\_065065863\_711-model4.36 | -11.4924 |
|  | XP\_065065863\_711-model4.37 | -11.4924 |
|  | XP\_065065863\_711-model4.38 | -11.4924 |
|  | XP\_065065863\_711-model4.39 | -11.4924 |
|  | XP\_065065863\_711-model4.40 | -11.4924 |
|  | XP\_065065863\_711-model4.41 | -11.4924 |
| # cluster 5 - Size: 19 |  |  |
|  | XP\_065065863\_711-model5 | -11.4907 |
|  | XP\_065065863\_711-model5.1 | -11.4907 |
|  | XP\_065065863\_711-model5.2 | -11.4907 |
|  | XP\_065065863\_711-model5.3 | -11.4907 |
|  | XP\_065065863\_711-model5.4 | -11.4907 |
|  | XP\_065065863\_711-model5.5 | -11.4907 |
|  | XP\_065065863\_711-model5.6 | -11.4907 |
|  | XP\_065065863\_711-model5.7 | -11.4907 |
|  | XP\_065065863\_711-model5.8 | -11.4907 |
|  | XP\_065065863\_711-model5.9 | -11.4907 |
|  | XP\_065065863\_711-model5.10 | -11.4907 |
|  | XP\_065065863\_711-model5.11 | -11.4907 |
|  | XP\_065065863\_711-model5.12 | -11.4907 |
|  | XP\_065065863\_711-model5.13 | -11.4907 |
|  | XP\_065065863\_711-model5.14 | -11.4907 |
|  | XP\_065065863\_711-model5.15 | -11.4907 |
|  | XP\_065065863\_711-model5.16 | -11.4907 |
|  | XP\_065065863\_711-model5.17 | -11.4907 |
|  | XP\_065065863\_711-model5.18 | -11.4907 |
| # cluster 6 - Size: 14 |  |  |
|  | XP\_065065863\_711-model6 | -11.4791 |
|  | XP\_065065863\_711-model6.1 | -11.4791 |
|  | XP\_065065863\_711-model6.2 | -11.4791 |
|  | XP\_065065863\_711-model6.3 | -11.4791 |
|  | XP\_065065863\_711-model6.4 | -11.4791 |
|  | XP\_065065863\_711-model6.5 | -11.4791 |
|  | XP\_065065863\_711-model6.6 | -11.4791 |
|  | XP\_065065863\_711-model6.7 | -11.4791 |
|  | XP\_065065863\_711-model6.8 | -11.4791 |
|  | XP\_065065863\_711-model6.9 | -11.4791 |
|  | XP\_065065863\_711-model6.10 | -11.4791 |
|  | XP\_065065863\_711-model6.11 | -11.4791 |
|  | XP\_065065863\_711-model6.12 | -11.4791 |
|  | XP\_065065863\_711-model6.13 | -11.4791 |
| # cluster 7 - Size: 2 |  |  |
|  | XP\_065065863\_711-model7 | -11.4203 |
|  | XP\_065065863\_711-model7.1 | -11.4203 |
|  |  |  |
